# Nutritional Status of Pre-school Children and Determinant Factors of Autism: A Case-Control Study

**DOI:** 10.3389/fnut.2021.627011

**Published:** 2021-02-19

**Authors:** Hana Alkhalidy, Amal Abushaikha, Khadeejah Alnaser, Mohammad D. Obeidat, Islam Al-Shami

**Affiliations:** ^1^Department of Nutrition and Food Technology, Faculty of Agriculture, Jordan University of Science and Technology, Irbid, Jordan; ^2^Department of Animal Production, Faculty of Agriculture, Jordan University of Science and Technology, Irbid, Jordan; ^3^Department of Clinical Nutrition and Dietetics, Faculty of Allied Health Science, The Hashemite University, Zarqa, Jordan

**Keywords:** autism spectrum disorder, nutritional status, children, maternal, determinant factors, Jordan

## Abstract

Autism Spectrum Disorder (ASD) is a neurodevelopmental disorder triggered by several factors, including those of genetic and environmental nature. ASD can alter communication, behavior, and children's nutritional status, placing them at high risk for nutritional imbalances. Therefore, this study aims to assess preschool autistic children's nutritional status as compared to that of Typically Developing (TD) children of the same age. The study also revealed some of the ASD risk factors among the Jordanian population. It included 52 ASD and 51 TD children (3–6 years), and considered sociodemographic, obstetric, and nutritional factors of the two groups, stratified by gender. Nutritional status was evaluated through a comprehensive questionnaire, 3-day food record, and anthropometric and biochemical measurements. Differences between groups were identified using the chi-square and independent-sample *t*-test. The logistic regression model was used after the adjustment of confounders to detect an autistic child's determinants. The study showed little difference between ASD and TD children with respect to nutrients' intake inadequacy and biochemical-nutritional deficiencies, but did reveal gender-based differences. Autistic girls were at higher risk of inadequate carbohydrate intake, while autistic boys were at higher risk of inadequate vitamin E, vitamin K, and fluoride compared to TD children. More autistic children had been treated in neonatal care units after birth than had TD children. The regression analysis revealed that lower maternal education level (OR, 12.25; 95% CI, 1.18–126.91), vaginal delivery (OR, 0.273; 95% CI, 0.105–0.712), family history of autism (OR, 0.189; 95% CI, 0.059–0.612), and taking dietary supplements during pregnancy (OR, 4.665; 95% CI, 1.158–18.79) were all determinants of ASD in children. In conclusion, maternal nutrition, postnatal conditions, and nutritional status might be contributors to ASD in children. Pre-school children are at high risk for developing nutritional deficiencies. It is therefore important to maintain optimal nutritional status in pregnant patients, and in children after delivery and during early childhood. Future studies that investigate the role of nutrient deficiencies and nutritional interventions in ASD are necessary. Also required are studies that focus on gender differences in the prevalence of ASD, types and severity of symptoms, and ASD nutrition-related problems.

## Introduction

Autism spectrum disorder (ASD) is a neurodevelopmental disorder characterized by lack of social and emotional interactions (1), repetitive behaviors, and restricted verbal and non-verbal linguistic skills (2). During the past several years, the number of ASD cases has increased, with a prevalence of 62 per 10,000 and a higher occurrence in boys compared to girls (ratio of 5:1) (3, 4). Autism is not well-understood and its occurrence is attributed to several environmental (5), genetic (6), neurological, gastrointestinal, and immunological factors (7). Many ASD children suffer from multiple nutrition-related issues such as maldigestion, malabsorption (8), abnormal fatty acid and amino acid metabolism, or multiple food intolerances (9). Also, ASD children display eating problems, food selectivity and increased refusal of new food items, which could place them at greater risk for nutritional excess or/and deficiencies (10, 11). Inadequacies of several nutrients were reported in ASD children were reported (12). As such, a comprehensive array of symptoms in the digestive tract in ASD children suggest the need for introducing nutritional interventions that could decreasing some ASD symptoms that may be associated with imbalanced nutrient intake and/or utilization (13–15).

Nutrition assessment is the first step in identifying nutrition-related problems and, thus, implementing a nutritional intervention to restore healthy nutritional status (16, 17). Data from the nutritional assessment domains showed differences in the levels of some nutrients and antioxidants when comparing ASD children to TD (Typically Developing) children from several countries (11, 18–21). Differences in the measured parameters suggest association with several factors, including health conditions present in the ASD children, nutrition care provided by the caregivers, and/or population-based factor (22–24). In addition, it is critical to evaluate nutritional status and identify children's nutrition-related problems early in life as it can have long-term impact on their health if left uncorrected (25). Therefore, this study evaluated the nutritional status of pre-school-aged ASD children using nutritional assessment domains (anthropometric measurements, dietary intake of macro-micronutrients, and the levels of common biochemical parameters) and compared them to that of TD children of the same age. These domains were compared according to gender. This study also identifies demonstrated some of ASD risk factors common in the Jordanian population.

## Materials and Methods

### Study Design and Participants

This case-control study of ASD children compared to their TD counterparts was conducted in Amman (Jordan), between July 2018 and February 2019. The study population included pre-school children aged 3–6 years and their mothers. The caregivers/parents signed the consent of participation with detailed information about the study's purpose, all measurements, and laboratory tests. Children were included in the study if they were previously diagnosed with ASD. Children with severe health issues and illnesses or those with a confirmed intake of supplements (vitamins and minerals) in the last 2 months were excluded from the study. The investigator visited 19 special needs centers in Amman, where most of these centers were at the time of the study. Fourteen centers agreed to take part in the study, however, after conducting thorough interviews with the centers' directors, only 8 centers had ASD children meeting the study inclusion criteria. The investigator met with the caregivers of 69 ASD children from these centers; 14 did not meet the inclusion criteria and 3 refused to participate in the study with a response rate of 94.6%. The number of ASD girls treated in the special needs centers was low. The maximum realizable number of ASD girls participated in this study. The final sample included 52 ASD cases (37 boys and 15 girls). For TD children, 51 children were recruited from two kindergartens in Amman (26 boys and 25 girls). Parents met with the principal investigator to complete questionnaires that gathered information about sociodemographics, maternal history, pregnancy and delivery, and child health and well-being, particularly surrounding their eating habits. Additional questions addressed the child's main ASD characteristics and symptoms. The study protocol was approved by the Institutional Review Board at Jordan University of Science and Technology (JUST) (Approval # 57/118/2018).

### Anthropometric Measurements

Weight and height were measured using an electronic scale attached to a height-measuring rod (AutoSike EBS-300RT digital scale with stadiometer, China). Weight was measured to the nearest 0.1 kg, and height was measured to the nearest 0.1 cm according to a standard procedure (26). The BMI was calculated using Quetelet's formula [(weight (kg)/height^2^ (m^2^)] (27). Children's height, weight, and BMI were subsequently plotted on their CDC clinical growth charts (children ages 2–20 years) (28). Children were classified as being obese (≥95th percentile), overweight (>85th and <95th percentile), normal (>5th to <85th percentiles), and underweight (<5th Percentile) according to CDC criteria (29). The z-scores of weight-for-age (WAZ), height-for-age (HAZ), and BMI-for-Age (BAZ) were calculated and classified according to the WHO criteria: Stunted with HAZ <-2, Underweight with WAZ <-2, Wasted with BAZ <-2, At risk of overweight, overweight and obese with BAZ >1, >2, and >3, respectively (30).

### Dietary Intake

The dietary intake of children was assessed using a 3-days food record (2 weekdays and 1 weekend) which is considered the method of choice for assessing nutrient intake among autistic children (31, 32). Through interviews, the dietitian used standard household measuring tools and food models to educate the parents about the portion size and help them estimate the children's consumption. The dietician analyzed dietary intake using a Computerized Nutrient Analysis Program “ESHA software” (version 10.63). Several Jordanian dishes and local foods were not registered in the ESHA database. Nutrient data for these items were derived from the food composition tables related to local foods in Jordan (3) and the Middle East (4), and were inserted into the database. Nutritional adequacy was determined by comparing the estimated intake with the Acceptable Macronutrient Distribution Range (AMDR) and Dietary Reference Intakes (DRIs), including recommended dietary allowances (RDAs) and adequate intakes (AIs) (33). The total energy requirement (TEE) for each child was calculated based on the WHO recommendations according to age, weight, height, gender and activity level (34). Reference values for macronutrients and micronutrients for children between 1 and 3 years and children between 4 and 6 years were drawn from the dietary guidelines for Americans (35). In this study, children's intake of a nutrient was considered inadequate if the mean intake was <67% of the RDA/AI (36–38).

### Biochemical Parameters Determination

Blood samples (5 ml) were collected from each participant in EDTA blood tubes and transferred to the JUST laboratory for subsequent analysis. Red Blood Cell (RBCs) and White Blood Cell (WBCs) counts were determined by the Coulter method (39). Hemoglobin (Hgb) levels were measured by the photometric method (40). Ferritin and 25-hydroxyl vitamin D were measured by an immunoassay analyzer (Access 2) using the Access 2 ferritin kit and the Access 2 25(OH) Vitamin D kit (Beckman Coulter, Brea, CA, USA, RRID:SCR_008940). For calcium determination, a photometric color test was done using clinical chemistry analyzer (Beckman Coulter AU analyzer, USA, RRID: SCR_008940). Alkaline Phosphatase (ALP) was evaluated using the kinetic change in color test as described by the International Federation for Clinical Chemistry (41). For analyzing inorganic phosphorous, a photometric UV test was used (42). The measured values for ASD and TD children were compared with the reference ranges obtained from the analytical laboratory at Jordan University of Science and Technology health center.

### Data Analysis

Data was analyzed using the Statistical Package for Social Sciences “SPSS software” (IBM SPSS Statistics for Windows, Version 21.0. Armonk, NY: IBM Corp, RRID: SCR_019096). Continuous variables were described using mean and standard deviation, and categorical variables were described using percentage. Chi-square (χ^2^) was performed to test the differences between ASD and TD children. An independent-samples *t*-test was performed to compare the mean and standard deviation of continuous variables between the two groups. Logistic regression models were used to detect determinants of having an autistic child by taking the TD children as the reference category. For each variable, the number of non-missing values are used. We specify the missing = listwise sub-command to exclude data if there is a missing value for any variable in the list. So, by default, missing values are excluded, and percentages are based on the number of non-missing values. Correlations are computed based on the number of pairs with non-missing data. For regression analysis, if values of any of the variables included in the analysis equation are missing, the entire case is excluded from the analysis. Multivariate-adjusted ORs with 95% CIs were estimated as increased risk for having an autistic child (OR > 1). Findings with a *p* < 0.05 were considered statistically significant.

## Results

### Participants' Characteristics

The final sample included 103 ASD and TD children with a mean age of (4.40 ± 0.80) and (4.18 ± 0.81), respectively (Data is not shown in tables). The general characteristics of the sample are shown in Table 1. A higher percentage of children were from male gender (61.2%), aged ≥5 years (42.7%), their family's average monthly income is >800 JD (45.6%). A significant difference shown between ASD and TD children; male gender is the dominant in ASD group (71.2%, *P* =0.036). The two groups differ significantly in their fathers' (*P* = 0.007) and mothers' (*P* = 0.042) occupation, with more ASD parents either unemployed or working in the governmental sector. ASD parents had a lower education level compared to TD parents. However, the difference between the groups was significant only for the mother's education level (*P* = 0.030).

**Table 1:** Comparison of general characteristics and family history between ASD and TD children.

**General Characteristics**	**Total**	**ASD *N* (%)**	**TD *N* (%)**	***p*-value**
**Gender**				
Female	40 (38.8)	15 (28.8)	25 (49.0)	0.036
Male	63 (61.2)	37 (71.2)	26 (51.0)	
**Age (years)**				
3>4	20 (19.4)	7 (13.5)	13 (25.5)	0.303
4>5	39 (37.9)	21 (40.4)	18 (35.3)	
5 ≥ 6	44 (42.7)	24 (46.2)	20 (39.2)	
**Father's occupation**				
Unemployed	1 (1.00)	1 (1.90)	0 (0.00)	0.007
Government sector	24 (23.3)	19 (36.5)	5 (9.80)	
Private sector	57 (55.3)	22 (42.3)	35 (68.6)	
Self-Employment	21 (20.4)	10 (19.2)	11 (21.6)	
**Father's education**				
Illiterate	1 (1.00)	1 (1.90)	0 (0.00)	0.149
Primary/Secondary school	34 (33.0)	21 (40.4)	13 (25.5)	
Bachelor's degree	68 (66.0)	30 (57.7)	38 (74.5)	
**Mother's occupation**				
Unemployed	74 (71.8)	36 (69.2)	38 (51.4)	0.042
Governmental sector	18 (17.5)	13 (25.0)	5 (27.8)	
Private sector	10 (9.70)	2 (3.80)	8 (80.0)	
Self-Employment	1 (1.00)	1 (1.90)	0 (0.00)	
**Mother's education**				
Illiterate	7 (6.80)	6 (11.5)	1 (2.00)	0.030
Primary/Secondary school	31 (30.1)	19 (36.5)	12 (23.5)	
Bachelor's degree	65 (63.1)	29 (51.9)	38 (74.5)	
**Family income**				
<350 JOD	10 (9.70)	8 (15.4)	2 (3.90)	0.167
350–800 JOD	46 (44.7)	21 (40.4)	25 (49.0)	
>800 JOD	47 (45.6)	23 (44.2)	24 (47.1)	

*ASD, autism spectrum disorder; TD, typically developing children; GI, Gastrointestinal; JOD, Jordanian Dinar*.

*Significant difference at p < 0.05. Chi-square (χ^2^) test*.

A comparison of some postnatal characteristics between ASD and TD children are summarized in Table 2. These characteristics included the family history of autism, neonatal care, breast feeding, if the child has ≥1 sibling or if he/she was the first child in his/her family, as well as his/her general appetite. These variables were analyzed and the difference between ASD and TD children was studied. Children's characteristics did not vary significantly between the two groups except for neonatal care; 13.5% of ASD children spent time in the neonatal unit, while none of the TD children did (*P* = 0.007). Some pregnancy-related factors are presented in Table 3; the results indicated that more ASD children were born through cesarean section compared to TD children (55.8 vs. 27.5%, *P* = 0.004). TD mothers were more committed to the intake of prenatal supplements (96.1 vs. 75.0%, *P* = 0.002) and folic acid (39.2 vs. 17.3%, *P* = 0.013) compared to ASD mothers.

**Table 2:** Comparison of children and postnatal characteristics between ASD and TD children.

**Characteristics**	**Total**	**ASD *N* (%)**	**TD *N* (%)**	***p*-value**
Siblings ≥1	53 (51.7)	26 (50.0)	27 (52.9)	0.765
First-born Child	41 (39.8)	20 (38.5)	21 (41.2)	0.778
Autistic person in family	23 (22.3)	14 (26.9)	9 (17.6)	0.258
Neonatal care	7 (6.80)	7 (13.5)	0 (0.00)	0.007
Breast-fed	82 (79.6)	40 (76.9)	42 (82.4)	0.494
**Appetite**				
High	28 (27.2)	13 (25.0)	15 (29.4)	0.565
Moderate	53 (51.5)	29 (55.8)	24 (47.1)	
Low	21 (20.4)	9 (17.3)	12 (23.5)	
Variable	1 (1.00)	1 (1.90)	0 (0.00)	

*ASD, autism spectrum disorder; TD, typically developing children*.

*Significant difference at p < 0.05. Chi-square (χ^2^) test*.

**Table 3:** Comparison of prenatal characteristics between ASD and TD children.

**Prenatal characteristics**	**Total**	**ASD *N* (%)**	**TD *N* (%)**	***p*-value**
**Pregnancy-related factors**				
Smoking	17 (16.5)	10 (19.2)	7 (13.7)	0.452
Disease during pregnancy	32 (31.1)	19 (36.5)	13 (25.5)	0.226
Prenatal supplements intake	88 (85.4)	39 (75.0)	49 (96.1)	0.002
Folic acid intake before pregnancy	29 (28.2)	9 (17.3)	20 (39.2)	0.013
**Type of delivery**				
Normal	60 (58.3)	23 (44.2)	37 (72.5)	0.004
Cesarean	43 (41.7)	29 (55.8)	14 (27.5)	

*ASD, autism spectrum disorder; TD, typically developing children*.

*Significant difference at p < 0.05. Chi-square (χ^2^) test*.

There were gender differences among ASD groups, however, these differences did not vary significantly for all the characteristics and symptoms except for speech therapy as a treatment type (Table 4). All ASD girls were treated by speech therapy compared to 78.4% of ASD boys (*P* = 0.050), and more boys received diet therapy compared to girls (24.3 vs. 13.3%, *P* = 0.379). The severity of ASD was more mild in boys (67.6%), and more moderate in girls (46.7%). Also, the ASD girls experienced fever more frequently during their first year of life. For ASD symptoms, girls (86.7%) were more affected by their mood than boys (75.7%), and the presence of ASD-related symptoms such as sleep disturbances, eating problems, and chewing difficulties which were also higher in girls compared to boys. Some Gastrointestinal (GI) symptoms were more prevalent in ASD boys, such as diarrhea, bloating, and vomiting. Also, ASD-related symptoms, including diarrhea, bloating, and hyperactivity, were more likely to be triggered by food in ASD boys compared to ASD girls. The results revealed that ASD girls (60.0%) resist new foods more than do ASD boys (48.6%).

**Table 4:** ASD main characteristics and symptoms stratified by gender.

**Category**	**Boys**	**Girls**	***p*-value**
**Severity of ASD**			
Mild	25 (67.6)	8 (53.3)	
Moderate	12 (32.4)	7 (46.7)	0.334
1st year fever-exposure	8 (21.6)	6 (40.0)	0.176
**ASD-related symptoms**			
Sleep disturbances	4 (10.8)	3 (20.0)	0.379
Eating problems	19 (51.4)	9 (60.0)	0.571
Chewing and swallowing	3 (8.10)	2 (13.3)	0.563
difficulties			
GI problems	16 (43.2)	7 (46.7)	0.822
**GI symptoms**			
Diarrhea	5 (20.8)	0 (0.00)	
Constipation	7 (29.2)	5 (50.0)	
Bloating	7 (29.2)	1 (10.0)	
Colic	0 (0.00)	2 (20.0)	
Vomiting	1 (4.20)	0 (0.00)	0.097
**Symptoms triggered by food**			
Diarrhea	9 (24.3)	2 (13.3)	0.379
Bloating	15 (40.5)	4 (26.7)	0.347
Hyperactivity	32 (86.5)	10 (66.7)	0.100
**Dietary characteristics**			
Following a diet	10 (27.0)	4 (26.7)	0.979
Breakfast intake	34 (91.9)	13 (86.7)	0.563
Lunch intake	37 (100)	15 (100)	–
Dinner intake	34 (91.9)	14 (93.3)	0.860
**Treatment**			
Behavioral therapy	32 (86.5)	11 (73.3)	0.256
Speech therapy	29 (78.4)	15 (100)	0.050
Diet therapy	9 (24.3)	2 (13.3)	0.379
Food selectivity	27 (73.0)	11 (73.3)	0.979
Influence of mood	28 (75.7)	13 (86.7)	0.379
Resistance to new food	18 (48.6)	9 (60.0)	0.458

*Significant difference at p < 0.05. Chi-square (χ^2^) test*.

### Anthropometric Measurements

The mean values for weight-for-age, stature-for-age, BMI-for-age, and z-scores are shown in Table 5. The results showed no significant differences in these parameters between ASD and TD groups. The mean values for these parameters were within the normal ranges. However, the average means for WAZ and HAZ were lower in TD boys compared to ASD boys, which may put more TD boys at risk of underweight and stunting than ASD boys. For BAZ, ASD boys had a higher mean, putting more of them at risk of overweight, compared to TD boys. On the other, ASD girls had lower means for WAZ, HAZ, and BAZ than TD girls. The findings from this study may point toward higher risk of undernutrition for ASD girls compared to ASD boys such as underweight (WAZ: −0.350 ± 1.68 and −0.023 ±1.57, respectively), and stunting (HAZ: −0.620 ± 1.36 and −0.135 ± 1.73, respectively).

**Table 5:** Anthropometric data: percentiles of weight-for-age, height-for-age, BMI-for-age, and Z-scores of ASD and TD children stratified by gender.

**Anthropometrics**	**Boy**			**Girl**		
	**ASD**	**TD**	***p*-value**	**ASD**	**TD**	***p*-value**
	**←mean ± SD →**			**←mean ± SD →**		
Weight-for-age	49.8 ± 34.2	39.9 ± 32.1	0.250	43.7 ± 38.8	57.4 ± 31.3	0.228
Stature-for-age	45.4 ± 37.3	39.3 ± 34.2	0.508	33.5 ± 33.8	51.9 ± 29.2	0.076
BMI-for-age	58.4 ± 31.8	57.3 ± 27.2	0.889	60.3 ± 32.5	66.0 ± 26.7	0.550
WAZ	−0.023 ± 1.57	−0.340 ± 1.25	0.396	−0.350 ± 1.68	0.220 ± 1.37	0.260
HAZ	−0.135 ± 1.73	−0.490 ± 1.69	0.418	−0.620 ± 1.36	−0.090 ± 1.28	0.231
BAZ	0.320± 1.36	0.190 ± 0.98	0.692	0.210 ± 1.73	0.620 ± 1.01	0.361
**BMI percentile**	**Boy**			**Girl**		
	**ASD**	**TD**	***p*****-value**	**ASD**	**TD**	***p*****-value**
	**←*N* (%) →**			**←*N* (%) →**		
Underweight	3 (8.10)	1 (3.80)	0.175	2 (13.3)	0 (0.00)	0.184
Normal weight	25 (67.6)	20 (76.9)		8 (53.3)	17 (68.0)	
Overweight	2 (5.40)	4 (15.4)		1 (6.70)	4 (16.0)	
Obese	7 (18.9)	1 (3.80)		4 (26.7)	4 (16.0)	

*ASD, Autism Spectrum Disorder; TD, Typically Developing; WAZ, Weight-For-Age Z-score; HAZ, Height-For-Age Z-score; BAZ, BMI-For-Age Z-score; BMI, Body Mass Index*.

*Significant difference at p < 0.05. Independent-samples t-test, chi-square (χ^2^) test*.

For BMI classifications, insignificant differences indicated between both ASD and TD groups in each gender category (*P* = 0.175 for boys and *P* = 0.184 for girls) as presented in Table 5. The prevalence of underweight was higher among ASD boys and girls (8.10 and 13.3%) compared to TD boys and girls (3.80 and 0.00%, respectively). Further, more ASD children were obese (18.9% in boys and 26.7% in girls) compared to TD children (3.80% in boys and 16.0% in girls). Both underweight and obesity were higher in ASD girls compared to ASD boys.

### Dietary Intake

Food records were collected to assess the nutritional status and identify dietary factors associated with autism incidence among our study population. A comparison of the inadequate daily energy and macronutrient intake between ASD and TD children is presented in Table 6. The 3-day food record showed that ASD girls had a significantly higher inadequacy from carbohydrate intake than TD girls (26.7 vs. 4.0%, *P* = 0.036). Other macronutrients intake did not vary significantly between the two groups. Nonetheless, more ASD boys did not meet the requirements for the daily energy and protein intake (27.8 and 19.4%, respectively) compared to TD boys (19.2 and 11.5%, respectively). The inadequate intake of carbohydrates and fats was seen in ASD girls more than ASD boys, who showed a higher percentage of inadequate protein intake.

**Table 6:** Comparison of the inadequate daily energy and macronutrient intake between ASD and TD children stratified by gender.

**Daily Macronutrients intake**	**Reference range**		**Boy**			**Girl**		
	**1–3 years**	**4–8 years**	**ASD *N* (%)**	**TD *N* (%)**	***p*-value**	**ASD *N* (%)**	**TD *N* (%)**	***p*-value**
Calories (kcal)			10 (27.8)	5 (19.2)	0.553	1 (6.70)	5 (20.0)	0.381
Protein (g/kg)	13	19	7 (19.4)	3 (11.5)	0.499	0 (0.00)	3 (12.0)	0.279
Carbohydrates (g)	130	130	6 (16.7)	6 (23.1)	0.528	4 (26.7)	1 (4.00)	0.036
Fat (g)	30–40	25–35	20 (55.6)	17 (65.4)	0.600	9 (60.0)	19 (76.0)	0.311

*ASD, Autism Spectrum Disorder; TD, Typically Developing Child*.

*Significant difference at p < 0.05. Chi-square (χ^2^) test*.

Micronutrient intake was analyzed to complete the assessment of the children's nutritional status. Gender differences among our study population between both ASD and TD groups were studied for inadequate intake of vitamins (Table 7) and minerals (Table 8). ASD boys exhibit significantly higher percentages of inadequacy of vitamin E (94.4 vs. 73.1%, *P* = 0.015) and vitamin K (86.1 vs. 61.5%, *P* = 0.026) than do TD boys. The results showed higher rates of inadequacy of vitamin B_2_ and vitamin B_6_ intake among ASD boys compared to TD boys, while more ASD girls failed to meet the daily needs of pantothenic acid, and biotin compared to TD girls. All children, ASD and TD, fell short of the requirements for vitamin A, vitamin D, vitamin B_12_, and folate.

**Table 7:** Comparison of the inadequate vitamin intake between ASD and TD children stratified by gender.

**Daily vitamins intake**	**Reference range**		**Boy**			**Girl**		
	**1–3 years**	**4–8 years**	**ASD *N* (%)**	**TD *N* (%)**	***p*-value**	**ASD *N* (%)**	**TD *N* (%)**	***p*-value**
Vitamin C (mg)	15.0	25.0	2 (5.60)	2 (7.70)	1.000	1 (6.70)	2 (8.00)	1.000
Vitamin B_1_ (mg)	0.50	0.60	1 (2.80)	1 (3.80)	1.000	0 (0.00)	1 (4.00)	1.000
Vitamin B_2_ (mg)	0.50	0.60	3 (8.30)	1 (3.80)	0.633	1 (6.70)	2 (8.00)	1.000
Niacin (mg)	6.00	8.00	0 (0.00)	1 (3.80)	0.119	0 (0.00)	0 (0.00)	0.502
Pantothenic Acid (mg)	2.00	3.00	20 (55.6)	15 (57.7)	1.000	12 (80.0)	15 (60.0)	0.298
Vitamin B_6_ (mg)	0.50	0.60	3 (8.30)	0 (0.00)	0.258	2 (13.3)	3 (12.0)	1.000
Biotin (mcg)	8.00	12.0	21 (58.3)	17 (65.4)	0.608	11 (73.3)	15 (60.0)	0.502
Folate (mcg)	150	200	36 (100)	26 (100)	ND	15 (100)	25 (100)	ND
Vitamin B_12_ (mcg)	0.90	1.20	36 (100)	26 (100)	ND	15 (100)	25 (100)	ND
Vitamin A	300	400	36 (100)	26 (100)	ND	15 (100)	25 (100)	ND
Vitamin D (mcg)	15.0	15.0	36 (100)	26 (100)	ND	15 (100)	25 (100)	ND
Vitamin E (mg)	6.00	7.00	34 (94.4)	19 (73.1)	0.015	14 (93.3)	22 (88.0)	1.000
Vitamin K (mcg)	30.0	55.0	31 (86.1)	15 (61.5)	0.026	14 (93.3)	22 (88.0)	1.000

*ASD, Autism Spectrum Disorder; TD, Typically Developing Child; ND, Not Detected; All children in the study population were inadequate*.

*Significant difference at p < 0.05. Chi-square (χ^2^) test*.

**Table 8:** Comparison of the inadequate mineral intake between ASD and TD children stratified by gender.

**Minerals**	**Reference range**		**Boy**			**Girl**		
	**1–3 years**	**4–8 years**	**ASD *N* (%)**	**TD *N* (%)**	***p*-value**	**ASD *N* (%)**	**TD *N* (%)**	***p*-value**
Calcium (mg)	700	1,000	22 (61.1)	13 (50.0)	0.443	10 (66.7)	16 (64.0)	1.000
Iron (mg)	7.0	10.0	1 (2.80)	2 (7.70)	0.567	3 (20.0)	2 (8.00)	0.345
Magnesium (mg)	80.0	130	36 (100)	26 (100)	ND	15 (100)	25 (100)	ND
Phosphorus (mg)	460	500	36 (100)	26 (100)	ND	15 (100)	25 (100)	ND
Zinc(mg)	3.00	5.00	36 (100)	26 (100)	ND	15 (100)	25 (100)	ND
Copper (mg)	0.34	0.44	1 (6.70)	4 (16.0)	0.037	0 (0.00)	3 (11.5)	0.388
Fluoride (mg)	0.70	1.00	36 (100)	23 (88.5)	0.037	15 (100)	25 (100)	ND
Selenium (mcg)	20.0	30.0	36 (100)	26 (100)	ND	15 (100)	25 (100)	ND

*ASD, Autism Spectrum Disorder; TD, Typically Developing Child; ND, Not Detected; All children in the study population were inadequate*.

*Significant difference at p < 0.05. Chi-square (χ^2^) test*.

As shown in Table 8, all children failed to meet their requirements from magnesium, phosphorus, zinc, and selenium. More ASD boys (61.1%) and girls (66.7%) failed to meet the daily requirements for calcium compared to TD boys and girls (50.0 and 64.0%). Also, more ASD girls (20.0%) had an inadequate iron intake than did TD girls (8.0%). However, these differences were not statistically significant. Boys showed a significant difference in fluoride; all ASD boys fell short of the fluoride requirements which was significantly higher when compared to TD boys (100 vs. 88.5%, *P* = 0.037). On the other hand, copper consumption among our study population was nearly better; frequency of inadequate copper intake was low among all groups; it was significantly lower among ASD boys than TD boys (6.70 vs. 16.0%, *P* = 0.037).

### Biochemical Parameters Determination

Biochemical analysis was performed to identify the differences between ASD and TD children. Values for each child were compared to the established reference values for each individual parameter to identify the abnormal results and the frequency of deficiency among the study population. Biochemical and nutrition-related, parameter levels are shown in Table 9 and Supplementary Table 1. CBC analysis showed that ASD boys had a significantly higher deficiency in MCHC (14.3 vs. 0.0%, *P* = 0.044) and HCT (60.0 vs. 11.5%, *P* < 0.001) than did TD boys, with a lower mean of serum HCT (36.6 ± 2.89 vs. 38.7 ± 2.03, *P* = 0.002). ASD girls had a significantly lower deficiency in MCV compared to TD girls (40.0 vs. 72.0%, *P* = 0.046). More ASD boys were deficient in HCT, MCV, MCH, and MCHC compared to ASD girls, and only ASD boys (5.7%) had a deficiency in HGB when compared to all other groups (0.0%). Children from the two groups had normal RBC levels. Although not significant, the mean RBC level was lower in ASD children than TD children, and lower in ASD girls compared to ASD boys. Whereas, there was no deficiency in RDW-CV in any of the groups, the mean RDW-CV was significantly higher in ASD boys when compared to TD boys (14.0 ± 1.31 vs. 13.1 ± 0.61, *P* = 0.004). In contrast, the majority of ASD and TD children had a deficiency in MCH; insignificantly higher in ASD girls than TD girls (60 vs. 84%). The deficiency in serum ferritin was more common among ASD boys (71.4%) and girls (46.7%) than in TD boys and girls (50.0 and 20.0%, respectively), but the difference was insignificant between ASD and TD children when not separated by gender. Ferritin deficiency was higher in boys from the two groups than in girls. Children were not deficient in serum levels for phosphorus, ALP, or calcium except for ASD boys, 2.9% of whom were deficient in calcium. Most of the children from the two groups were deficient in vitamin D_3_; though the difference was not significant vitamin D_3_ deficiency was more common among the TD boys and girls compared to ASD boys and girls.

**Table 9:** Comparison of the biochemical and nutritional deficiencies between ASD and TD children stratified by gender.

**Biochemical test**	**Reference range**	**Boy**			**Girl**		
		**ASD**	**TD**	***p*-value**	**ASD**	**TD**	***p*-value**
		**Deficiency *N* (%)**	**Deficiency *N* (%)**		**Deficiency *N* (%)**	**Deficiency *N* (%)**	
WBC (10^∧^9/L)	4.00–11.00	0 (0.00)	2 (7.70)	0.178	0 (0.00)	2 (8.00)	0.519
RBC (10^∧^12/L)	3.50–5.50	0 (0.00)	0 (0.00)	ND	0 (0.00)	0 (0.00)	ND
HGB (g/dL)	11.0–16.0	2 (5.70)	0 (0.00)	0.503	0 (0.00)	0 (0.00)	ND
HCT (%)	37.0–54.0	21 (60.0)	3 (11.5)	<0.001	4 (26.7)	2 (8.00)	<0.001
MCV (fl)	80.0–100	25 (71.4)	17 (65.4)	0.781	6 (40.0)	18 (72.0)	0.046
MCH (pg)	27.0–34.0	29 (82.9)	23 (88.5)	0.720	9 (60.0)	21 (84.0)	0.135
MCHC (d/dL)	32.0–36.0	5 (14.3)	0 (0.00)	0.044	2 (13.3)	0 (0.00)	0.135
RDW-CV (%)	11.0–16.0	0 (0.00)	0 (0.00)	ND	0 (0.00)	0 (0.00)	ND
Ferritin (ng/ml)	12.0–300	25 (71.4)	13 (50.0)	0.113	7 (46.7)	5 (20.0)	0.091
Calcium (mmol/L)	2.00–2.60	1 (2.90)	0 (0.00)	1.000	0 (0.00)	0 (0.00)	ND
25(OH)D (mg/ml)	30.0–100	33 (94.3)	25 (96.2)	1.000	14 (93.3)	25 (100)	0.375
Phosphorus (mmol/L)	0.80–1.50	0 (0.00)	0 (0.00)	ND	0 (0.00)	0 (0.00)	ND
ALP (U/L)	93.0–309	0 (0.00)	0 (0.00)	ND	0 (0.00)	0 (0.00)	ND

*ASD, Autism Spectrum Disorder; TD, Typically Developing Child; 25(OH)D, 25-Hydroxy Vitamin D; ND, Not Detected; All children in the study population were adequate*.

*Significant difference at p < 0.05. Chi-square (χ^2^) test*.

### Determinants of Autism Among Pre-school Children

Table 10 shows the adjusted association between some variables and autism presence using the stepwise selection method. Entry testing was based on the significance of the score statistic, and removal testing was based on the probability of a likelihood-ratio statistic based on conditional parameter estimates. Maternal education level, child's delivery method, family history of autism, and taking nutritional supplements during pregnancy were all predictors for having an autistic child. Results showed that illiterate mothers were 12 times more likely to have an autistic child than mothers who attained university education levels (OR = 12.25; CI 95% 1.18, 126.91), while mothers with lower education levels (i.e., primary and secondary school) were 3 times more likely to have an autistic child when compared to mothers with higher education levels (OR = 3.319; CI 95% 0.34, 40.62). Children who were delivered vaginally were less likely to be autistic than children delivered by cesareans births with an odds ratio = 0.273 (CI 95% 0.105, 0.712). Interestingly, families with autism history were less likely to have autistic children than were the families with no autism history, with an odds ratio = 0.189 (CI 95% 0.059, 0.612). Finally, mothers who took nutritional supplements during pregnancy were about 5 times more likely to have an autistic child than those who did not take supplements (OR = 4.665; CI 95% 1.16, 18.79).

**Table 10:** Predictors of Autism among our study population using logistic regression analysis‡.

**Variable**	**OR**	**95% CI**	***p*-value**
**Mother's education**			
Illiterate	12.25	1.18–126.91	0.036
Primary/secondary school	3.319	0.34–40.62	0.027
Bachelor's degree	Reference		
**Type of delivery**			
Normal	0.273	0.105–0.712	0.008
Cesarean	Reference		
**Family history of Autism**			
Yes	0.189	0.059–0.612	0.005
No	Reference		
**Maternal supplements**			
Yes	4.665	1.158–18.79	0.030
No	Reference		

‡ *Presented data are adjusted for all the demographics and familial variables*.

*OR means odds ratio and 95% CI is: 95% confidence intervals. Stepwise selection method*.

## Discussion

Several genetic and environmental factors are extensively bound with ASD (43). Our study discusses some environmental and obstetric factors related to ASD among Jordanian children. Similar to previous studies conducted globally, we found a higher prevalence of ASD among boys compared to girls hence the high recruitment of male participants than girls. Male gender might be a determinant for autism. The risk of disease is 4 times higher in genetically susceptible males than females (44). Beggiato et al. (45) reported that girls were more likely to be underidentified by some diagnostic instruments for ASD, and thus, the lower rates of ASD diagnosis compared to boys.

In this study, lower education level among parents correlates with a higher risk of having an autistic child. The results were consistent with a comparative study between ASD and TD children in China (15). In India, lower education level among ASD mothers, but not fathers, was associated with higher ASD incidence in offspring. However, the difference was not significant (46). Conversely, another Chinese study reported higher parental education level for parents in the ASD group than in the TD group (47).

The current study supports findings of previous studies that show being the first child in the family, cesarean delivery (48), and treatment in the neonatal care unit were associated with increased risk of ASD (49). Other factors such as family history, did not statistically differ between the two groups in our study. Contrary to expectation, the final regression analysis of our results showed that familial history of autism, while significant was inversely related to the risk of ASD in the offspring. Our results contradicted previous studies that confirmed higher ASD risk with increased family incidence of ASD (50, 51). Further investigation is merited. This study did not reveal any significant differences between the ASD and TD children who were breastfed, though our study demonstrated a similar trend as that in an Indian study wherein the lack of breastfeeding was significantly higher among ASD children (46). Unexpectedly, Dodds (44) reported that breastfeeding at the time of discharge might be associated with increased ASD risk.

In our study, the mothers of ASD children reported a higher occurrence of disease during pregnancy than mothers of TD children. Although our results were not statistically significant, they align with those of a study in which increased presence during pregnancy of medical conditions such as anemia, heart, pulmonary and renal diseases were associated with an increased risk of ASD in children (44). In line with previous studies (48, 52), smoking during pregnancy was not significantly associated with ASD. However, a higher percent of ASD children were born to smoking mothers in Jordan, which might correlated instead with socioeconomic status (53).

Another obstetric factor relating to decreased risk of autism is the intake of folic acid and other maternal supplements. Levine et al. (50) reported that folic acid and/or multivitamin intake during pregnancy correlates with a lower risk of ASD. The rationale behind this decrease in ASD risk might be the correction of nutritional deficiencies, and folate deficiency in particular. Mothers of ASD children might have serum autoantibodies directed at the folate receptor alpha (FRα) on the placental and the blood-brain barriers, causing an impairment in transferring folate to the fetus. Thus, folic acid supplementation plays a role in reducing ASD severity (54). Calcium supplementation in preparation for pregnancy coincided with a lower risk of ASD among Chinese children (47). Interestingly, the final analysis of our results showed that the mothers who took nutritional supplements during pregnancy were about 5 times more likely to have an autistic child than those who did not take supplements. Raghavan et al. (55) reported that adequate intake of supplements during pregnancy is linked with a decreased risk of ASD. However, both the inadequate intake and excessive intake are related to increased ASD risk in the offspring.

Some ASD-related symptoms observed in this study such as sleep disturbances, mood swings, and GI abnormality agrees with the literature (46). The most common GI abnormalities in our study were constipation, followed by bloating and diarrhea. Similarly, 22.1% of ASD children in Italy suffered from constipation, followed by painful bowel movements and cramps. These GI abnormalities might be associated with an increased risk of sleeping disturbances (56). ASD girls in our study were affected by mood and sleep disturbances more than boys, though the difference was not statistically significant. These results are consistent with previous research that indicates more anxiety, depression, and sleep abnormalities among ASD girls than TD boys (57). Another ASD-related characteristic that was significantly higher among ASD children than TD children (data not shown) is increased food selectivity and resistance to trying new foods. These behaviors were similar to ASD children's behaviors in the United States (58) and China (59). Most ASD children in our study were regularly taking their main meals, as opposed to 80% of ASD children who were skipping their regular meals in a study of Indian children (60).

In our study, there were higher WAZ, HAZ, and BAZ among ASD boys compared to TD boys. In Egypt, similar results were found among ASD children compared to TD children (61). Our study's results showed ASD girls to have lower WAZ, HAZ and BAZ compared to TD girls, which is consistent with the results from a study of ASD children in China (15, 59). Barnhill (21) and Malhi (12) reported no significant differences between ASD and TD children in weight, height, and BMI in the United States and India. Our results showed a higher prevalence of malnutrition, including underweight and overweight, in ASD children compared to TD children. Correspondingly, in Spain, ASD children had a significantly higher prevalence of underweight than TD children (11). Likewise, Egan et al. reported that the prevalence of overweight and obesity among ASD children was higher than population norms (62). Contrary to our study, one study found a lower prevalence of obesity among ASD children than TD children in China (15). Our results might be explained by increased consumption of unhealthy snacks and artificial sweeteners, and the decreased consumption of vegetables among ASD children in Jordan (data from the 3-day food record). Sleep disturbance, irregular eating habits, low physical activity, and psychopharmacologic medicine might also contribute to the observed increase in overweight or obesity in ASD children (63).

Although most children in the current study did meet their daily energy requirements, more ASD boys and TD girls failed to meet the daily energy needs than did other groups. Previous studies from China and the United States reported that ASD children had lower intakes of energy and all macronutrients than TD children (15, 64). We also found that ASD girls failed to meet the requirements for carbohydrate intake more frequently than did TD girls. The same intake pattern was found among children in Spain (11). In Egypt, more ASD children failed to meet the recommendations for protein intake than did TD children (61); we observed this among ASD boys in this study. More TD children in our study did not meet the total fat intake requirements compared with ASD children. Similarly, another study found that more ASD children had excessive fat intake than TD children (11).

Most children's intake from vitamin E and K was below the requirements, it being even lower among ASD children. Previous studies agreed that most children failed to meet vitamin E requirements (21), and that vitamin K intake was inadequate among ASD children (58). But, in Spain, most children had an adequate intake of vitamin E and vitamin K, with ASD children having a higher intake (11). ASD boys in our study had lower intake of vitamin B_2_ and B_6_. Our results align with previous studies among ASD children in the United States and Spain (11, 21, 64). ASD girls in our study had a lower intake of biotin, which is consistent with observations of a previous study in Spain (11). Girls also had a lower intake of vitamin B_6_ In contrast with a previous study that reported a higher intake of vitamin B_6_ among ASD children (11). All children had an inadequate intake of vitamin A in this study. This inadequacy of vitamin A intake is higher relative to another study, as nearly half of ASD and TD children had inadequate vitamin A intake (15). Other studies showed that ASD children exhibited lower intake of vitamin A than TD children (11, 21). All subjects in our study had inadequate vitamin D intake. The results were consistent with a study in which 2% of ASD children had adequate vitamin D intake compared to 0% among TD children (21). A lower percentage of inadequacy was found among children in Spain, but it was insignificantly higher among TD children (11). Several previous studies pointed to the lower intake of folic acid and vitamin B_12_ among ASD children (12, 21, 61, 64); however, we found that all children had inadequate intake of these two vitamins.

In this study, we found inadequate mineral intake (including magnesium, phosphorus, zinc, and selenium) in all children, with no difference between ASD and TD children. While there was no difference in the intake of zinc and phosphorus between the two groups in Egypt, magnesium, and selenium intake was lower among the ASD group (61). Conversely, ASD children in Spain had a higher zinc and magnesium intake compared to TD children (11). ASD children in the United States had a lower intake of zinc and a higher intake of selenium, which was inadequate in most children (21). Another study in the United States reported that ASD boys consumed less phosphorus and selenium compared to their healthy counterparts (64). While magnesium inadequacy was shared among all groups (10), it was higher among TD children (58). Our study further showed that children had inadequate fluoride intake similar to Spanish children (11). Our results showed a higher copper inadequacy among TD children, different from the copper intake among Indian children (12). As expected from a previous study (10), our study showed that calcium inadequacy was common among children, with a higher prevalence among ASD than TD children. Previous studies reported a lower intake of calcium and iron among ASD children (21, 61, 64). Neumeyer and colleagues reported that the inadequate intake of calcium, phosphorus, and protein among ASD boys was significantly related to lower bone mineral density (64) that may increase the risk of fractures among ASD children (65). Our study showed a higher inadequacy of iron intake among ASD compared to TD girls, though the result was insignificant. We also found higher inadequacy of iron intake among TD boys compared to ASD boys. In Spain, ASD children also exhibited higher intake of iron compared to TD children (11).

The differences in nutrient intake and nutritional adequacies among ASD and TD children from different countries might be affected by the dietary assessment tool used in the study (58). These differences can also be related to the amount of food intake and limited food choices among ASD children. For example, in India, 79% of ASD children had improper feeding behavior, such as selecting certain food items or having inadequate intake (12). This inadequate intake among children might be correlated with nutritional inadequacies (10). In our study, ASD children consumed few sources of vitamins and minerals, particularly seafood, eggs, and vegetables. A previous study found that the limited consumption of fruits, vegetables, meat, and poultry among ASD children was related to lower intake of B vitamins and micronutrients (60).

As HGB, HCT and MCV are lower among pre-school children, and specifically in ASD children (66), it was essential to assess these levels among ASD children in Jordan. Our results agreed with a previous study that indicated lower HCT among ASD children compared to TD children (66, 67). In our study, there was a minimal HGB deficiency among ASD boys. Similarly, in China only 2.08% of ASD children below age 6 exhibited HGB deficiency, while it was absent among older children (15). In contrast, other studies reported a significantly lower HGB level among ASD as opposed to TD children. They reported lower MCV among ASD children as a group, while this study found this in ASD boys, but not ASD girls (66, 67). Our results indicated that the mean level of WBC was lower among ASD than TD children, and the deficiency was higher among TD children; however, the difference was not significant in our study and previous studies (68, 69). ASD boys had a significantly higher mean for RDW than did TD boys. The results were consistent with a previous study among ASD children in Turkey (68). Other studies have indicated higher levels of RDW among TD children (67, 69). Our study findings showed that ferritin deficiency was higher among ASD children compared to TD children, and higher in ASD boys than ASD girls. These results among our study population were similar to those among children in the United States (67); indicating that the risk of iron deficiency and iron-deficiency anemia among ASD children is higher (20). Low ferritin levels among ASD children might be related to lower intake of dietary iron that results from restricted food choices (56).

In our study, calcium deficiency was present among 2.9% of ASD boys only. This result correspond with observations by Adams (20) whose study reported calcium in the serum of ASD and TD children in the normal range. Likewise, Neumeyer (64) found serum calcium level to be the same among the two groups. However, ASD children in Egypt had a lower serum level of calcium than the TD group (61). Nearly all children in our study presented with vitamin D_3_ deficiency, serum levels being lower than 30 mg/ml. This is a higher percentage compared to previous studies in the United States and Egypt in which 62 and 57% of children (respectively), had vitamin D deficiency (54, 70). There was no significant difference in the severity of vitamin D_3_ deficiency between ASD and TD children, but the incidence was higher among the TD girls compared to ASD girls. Conversely, studies reported a lower serum level of vitamin D_3_ among ASD children compared with TD children (59). Notably, Saad and colleagues reported that the level of vitamin D deficiency is associated with the severity of ASD symptoms (70). Vitamin deficiency among ASD children might be caused by inadequate dietary vitamin D intake due to the picky eating behavior among ASD children (59).

## Conclusion

Limitations of our study include the season that blood was drawn (winter, between January and February), affecting serum 25(OH)D levels because of the lack of sunlight exposure. However, vitamin D_3_ deficiency has become widespread for the rise in urbanized lifestyle and decreased sunshine exposure. And although the 3-day record was favorable as an assessment tool for children's dietary intake, the risk of underestimation or overestimation of certain food items is present. Nonetheless, our study effectively indicated the strong correlation between some environmental factors and the risk of ASD. Mothers' nutritional status during pregnancy and postnatal conditions could be strong contributors to autism spectrum disorder. Hence, maternal nutrition should be considered essential in trying to reduce the risk of ASD. Our assessment of children's nutritional status indicated that malnutrition, including underweight, overweight, obesity, and inadequate nutrient intake, was present in ASD and TD children. Planned intervention is needed to prevent malnutrition and ensure adequate nutrient intake in this age group, aiming to prevent any future developmental issues. The similarity in nutritional deficiencies between ASD and TD children may indicate that nutrition is not the main contributor to ASD. However, it might be part of a complex interplay between genetics, maternal, and environmental factors that leads to ASD among children. Moreover, the correction of these deficiencies might be necessary to reduce the severity of ASD and its related symptoms. We recommend influencing food selectivity and refusal among ASD children by starting with the family's favorite food items and considering the child's sensitivity to texture, smell, and flavor preferences. An individualized diet plan should also be prescribed for each autistic child according to the present ASD-related symptoms. The current study also revealed that ASD males and females might differ in the prevalence of the disease, type and severity of symptoms, and ASD nutrition-related problems. More studies are essential to explore the role of gender as a determinant factor for the severity of ASD and its related syndrome. Also, further studies are still needed to understand better the role of nutrients and other determinants of ASD.

## Data Availability Statement

The raw data supporting the conclusions of this article will be made available by the authors, without undue reservation.

## Ethics Statement

The studies involving human participants were reviewed and approved by the Institutional Review Board at Jordan University of Science and Technology, Irbid, Jordan. Written informed consent to participate in this study was provided by the participants' legal guardian/next of kin.

## Author Contributions

HA: study, concept and design, interpretation of data, critical revision of the manuscript for important intellectual content, obtained funding, administrative, technical support, and study supervision. AA: conducted the study, data collection and interpretation, and drafting of the manuscript. KA: data interpretation and critical revision of the manuscript for important intellectual content. MO: study design, administrative and material support and study supervision. IA-S: statistical analysis, interpretation of data, and critical revision of the manuscript for important intellectual content. All authors agree to be accountable for the content of the work.

## Conflict of Interest

The authors declare that the research was conducted in the absence of any commercial or financial relationships that could be construed as a potential conflict of interest.
