# Supplementary material for: Nutritional Status of Pre-school Children and Determinant Factors of Autism: A Case-Control Study
Source: Front Nutr. 2021 Feb 19;8:627011. doi: 10.3389/fnut.2021.627011 (PMC7933547; doi:10.3389/fnut.2021.627011)
Supplement: Supplementary file 1 [file Table_1.DOCX]

| Supplementary Table 1. Comparison of the mean values of the biochemical and nutritional levels between ASD and TD children stratified by gender | | | | | | | |
| --- | --- | --- | --- | --- | --- | --- | --- |
| **Biochemical Test** | **Reference Range** | **Boy** | |  | **Girl** | |  |
|  |  | **ASD** | **TD** | **p-value** | **ASD** | **TD** | **p-value** |
|  |  | **mean ± SD** | **mean ± SD** |  | **mean ± SD** | **mean ± SD** |  |
| **WBC (10ᵔ9/L)** | 4.00-11.00 | 8.34± 2.50 | 8.78±2.18 | **0.476** | 8.55± 20.3 | 9.23± 1.68 | **0.289** |
| **RBC (10ᵔ12/L)** | 3.50-5.50 | 4.87± 0.49 | 4.93± 0.28 | **0.637** | 4.73±0.35 | 4.85± 0.23 | **0.181** |
| **HGB (g/dL)** | 11.0- 16.0 | 12.6± 1.08 | 13.0± 0.69 | **0.060** | 13.05±0.68 | 13.0± 0.66 | **0.727** |
| **HCT (%)** | 37.0 -54.0 | 36.6± 2.89 | 38.7± 2.03 | **0.002** | 38.2± 2.07 | 38.4± 1.62 | **0.679** |
| **MCV (fl)** | 80.0 -100 | 76.2± 7.02 | 78.6± 3.07 | **0.098** | 80.4± 4.70 | 79.2± 2.80 | **0.329** |
| **MCH (pg)** | 27.0 -34.0 | 26.0± 3.12 | 26.5± 1.32 | **0.469** | 27.5± 1.93 | 26.8± 0.99 | **0.105** |
| **MCHC (d/dL)** | 32.0 -36.0 | 34.1± 1.55 | 33.6± 0.62 | **0.222** | 33.5± 2.76 | 33.8± 0.63 | **0.682** |
| **RDW-CV (%)** | 11.0 -16.0 | 14.0± 1.31 | 13.1± 0.61 | **0.004** | 13.6± 0.76 | 13.1± 0.89 | **0.80** |
| **Ferritin (ng/ ml)** | 12.0- 300 | 21.8± 17.6 | 25.5± 15.6 | **0.388** | 20.0± 20.5 | 27.8± 18.7 | **0.220** |
| **Calcium (mmol/L)** | 2.00 -2.60 | 2.36± 0.31 | 2.45± 0.08 | **0.195** | 2.42± 0.08 | 2.46± 0.08 | **0.152** |
| **25(OH)D (mg/ml)** | 30.0 -100 | 18.7± 6.72 | 20.2± 6.57 | **0.395** | 20.0± 7.15 | 18.1± 4.51 | **0.301** |
| **Phosphorus (mmol/L)** | 0.80 –1.50 | 1.66± 0.21 | 1.66± 0.16 | **0.995** | 1.31± 0.15 | 1.59± 0.15 | **0.200** |
| **ALP (U/L)** | 93.0 -309 | 207.4± 48.1 | 223.9± 57.7 | **0.222** | 281.5± 80.6 | 233.6± 39.2 | **0.167** |
| ASD: Autism Spectrum Disorder, TD: Typically Developing Child, 25(OH)D: 25-Hydroxy Vitamin D.  Significant difference at p <0.05. Chi-square (χ2) test | | | | | | |  |

Supplementary Material
